# Office workers’ perspectives on implementing active breaks supported by a digital intervention to alleviate spinal pain: a qualitative study

**DOI:** 10.1016/j.bjpt.2026.101634

**Published:** 2026-08-11

**Authors:** Carlos Tersa-Miralles, Cristina Bravo, Maria Masbernat-Almenara, Filip Bellon, Francesc Rubí-Carnacea, Esther Rubinat-Arnaldo

**Affiliations:** a Departament d'Infermeria i Fisioteràpia, University of Lleida, Lleida, Spain; b Grup de Recerca de Cures en Salut (GRECS), IRBLleida, Biomedical Research Institute Dr. Pifarré Foundation, Lleida, Spain; c Centro de Investigación Biomédica en Red de Diabetes y Enfermedades Metabólicas Asociadas (CIBERDEM), National Institute of Health Carlos III (ISCIII), Barcelona, Spain; d Grup d’Estudis Societat, Salut, Educació i Cultura (GESEC), University of Lleida, Lleida, Spain

**Keywords:** Exercise therapy, Occupational health, Office workers, Qualitative study, Sedentary behavior, Spinal pain

## Abstract

**Background:**

Work-related spinal pain is highly prevalent among office workers and contributes to disability and reduced productivity. Active breaks have shown benefits, but their integration into daily routines remains challenging.

**Objective:**

To explore office workers’ beliefs, expectations, and perceived barriers regarding a digital intervention designed to support active breaks at work for the management of work-related spinal pain.

**Methods:**

Seventeen office workers from the University of Lleida, all experiencing work-related spinal pain, participated in semi-structured interviews between June 2023 and February 2024. Data were analysed using Malterud’s systematic text condensation framework.

**Results:**

Three main themes emerged: work-related spinal pain, implementation of active breaks at work, and feasibility of a digital intervention in the workplace. Participants reported persistent spinal pain due to static postures and job stress, often leading to passive coping strategies. Although they perceived stretching and mobility exercises at work as beneficial, integrating them into daily routines was challenging due to heavy workloads and concerns about managerial perceptions. Employees preferred shorter, flexible breaks over longer interruptions. Digital interventions were viewed favourably for their convenience and accessibility, but personalised guidance from health professionals was considered crucial for adherence.

**Conclusion:**

Office workers expressed confidence in adopting a digital intervention for active breaks, supported by self-management strategies and physiotherapist guidance, to alleviate spinal pain. These findings highlight the importance of involving workers in designing workplace health programs tailored to their needs and preferences.

## Highlights

• Office workers experience persistent spinal pain due to static postures and stress.
• Active breaks of 3–5 min are feasible and may enhance adherence and effectiveness.
• High workload and managerial concerns hinder active‑break uptake.
• Digital interventions are valued for convenience and personalisation.
• Participant‑driven design enhances the relevance and sustainability of interventions.

## Introduction

Musculoskeletal disorders are one of the leading causes of disability for the population worldwide in terms of work disability, absenteeism and presenteeism.^1^ Office workers are particularly exposed to work-related spinal pain due to prolonged sitting, reduced physical activity and static postures, combined with psychosocial factors such as job strain.^2^

Ergonomic, educational and organizational strategies can help reduce musculoskeletal problems in office environments.^3^ While ergonomic interventions may be cost-effective and feasible,^4^ evidence suggests that a multicomponent approach including workplace exercise may be necessary to reduce work-related spinal pain.^5^

Workplace therapeutic exercise can reduce pain, although implementation remains heterogeneous.^6^ Misconceptions about pain and kinesophobia may reinforce prolonged sitting and avoidance behaviors, highlighting the need to integrate exercise-based coping strategies.^7^

Randomized controlled trials^8^^,^^9^ show that online tools are an effective and low-cost alternative for managing chronic musculoskeletal and spinal pain. However, further research is required to adapt these interventions to workplace contexts while considering workers’ preferences, self-management skills and presenteeism to support adherence.^10^

In workplace settings, participatory approaches may strengthen the feasibility of digital interventions by aligning them with workers’ routines and decision-making. Enabling workers to participate in the implementation process may help modify behavioral and lifestyle risk factors.^11^ Considering workers’ expectations and contexts could improve adherence and motivation towards workplace exercise strategies.^12^ Physiotherapists can support these processes by aligning exercise and self-management strategies with workplace demands.^13^

This qualitative study represents the first phase of a mixed-methods project to develop and evaluate a workplace intervention for spinal pain. The protocol has been published elsewhere.^14^ Understanding workers’ beliefs and expectations is essential to align exercise selection, frequency, and delivery with their needs and daily work routines, thereby improving adherence and ensuring the success of the intervention.

The study question for this qualitative study was:

What are office workers’ beliefs, expectations, and perceived barriers to adopting digital active breaks for spinal pain management at work?

## Material and methods

### Study design

This study used a descriptive qualitative design informed by phenomenological ideas. We selected this approach to describe participants’ experiences and meanings related to work-related spinal pain and the feasibility of active breaks in an applied occupational context, rather than to generate theory.^15^ Data were analyzed using Malterud’s systematic text condensation, a pragmatic analytic approach informed by phenomenological ideas.

The study was reported in accordance with the Standards for Reporting Qualitative Research (SRQR).^16^ A completed SRQR checklist indicating where each item is addressed in the manuscript is provided as Supplementary File 1.

### Participants

The participants in this study were recruited from among the technical, administrative, and service staff across five campuses of the University of Lleida. The recruitment process was initiated by establishing contact with the managers of the workers at each campus. This strategic approach ensured smooth communication between the research team and the potential participants who were contacted via institutional email.

A purposive sampling strategy was used to ensure diversity in occupational roles (administrative, technical, and service staff) and work profiles (public-facing and non-public-facing positions), with the aim of capturing a broad range of experiences related to work-related spinal pain and the feasibility of active breaks at work.

Eligible participants were workers experiencing persistent work-related spinal discomfort (lumbar, dorsal, and/or cervical) for more than three months. Spinal discomfort was defined based on participants’ self-reported symptoms associated with their work activities and daily occupational demands, rather than on clinical diagnosis or pain severity. Participants were required to be actively working at the time of recruitment (i.e., not on sick leave) and to spend over 80% of their working hours in sedentary tasks involving computer-based activities, public interactions, or telephone communications, in line with Parry et al.^17^

Although no new relevant themes emerged during ongoing data collection and preliminary analysis after fourteen interviews, recruitment continued until seventeen participants were included to ensure stability of the findings across different roles and work profiles. Data adequacy was considered in relation to the focused study aim, the specificity of the sample, and the consistency of themes across interviews, in line with the concept of information power.^18^

### Data collection

Data collection took place between June 2023 and February 2024. The semi-structured interviews were conducted by the principal investigator (CTM) and CB, both experienced in qualitative research and with no prior professional, clinical, or supervisory relationship with the participants. The interviews were thoughtfully scheduled during regular working hours and were conducted either within the participants' offices or in available rooms within the same faculty.

The majority of interviews lasted between 20 and 30 min (mean duration: 24 min). Although interviews could have been extended if necessary, this duration corresponded to the focused scope of the interview guide, which prioritized the key domains relevant to the research question. The informational depth generated through targeted questions and probing was sufficient for thematic analysis in relation to the study aim.

Interviews followed a semi-structured guide with open-ended questions and probes to explore participants’ perceptions and concrete experiences related to work-related spinal pain and the feasibility of incorporating active breaks.

To safeguard against potential data loss, interviews were dual-recorded using a dedicated voice recorder and computer-based recording. In addition, the interviewers took brief field notes to document contextual aspects of the setting, non-verbal cues, and initial analytical impressions, which supported both data interpretation and subsequent analytical discussions.

The interview guide was developed within the broader mixed-methods project and discussed within the research team to ensure alignment with the qualitative study aims. It was informed by relevant literature on work-related spinal pain and workplace exercise interventions. The full guide is provided in Supplementary File 2 and was previously published as supplementary material in the study protocol.^14^

The institutional virtual learning platform Sakai,^19^ used at the University, was proposed as a potential delivery channel for the intervention, as it is routinely used by staff in their day-to-day work.

### Data analysis

The interviews were audio-recorded and subsequently meticulously transcribed verbatim to ensure accurate representation. The research team employed Atlas.ti softwareTM for coding and data management.

To conduct qualitative data analysis, the study embraced the systematic text condensation framework proposed by Malterud,^15^ based on Giorgi's psychological phenomenological method.^20^ The analysis followed four steps: (1) all transcripts were read to gain an overall impression and note preliminary ideas; (2) meaning units related to beliefs, expectations, barriers, and preferences about spinal pain and active breaks were identified and marked in the text; (3) these units were condensed, coded, and grouped into preliminary categories; and (4) categories were synthesized into main themes and subthemes, supported by participant quotes.

Two researchers (CTM and CB) independently coded the transcripts and subsequently discussed and agreed on the coding and theme development to enhance analytical credibility. Researcher triangulation was achieved through independent coding by CTM and CB, followed by consensus discussions to refine codes and themes and to consider alternative interpretations of the data.

Reflexivity was addressed through ongoing discussions within the research team during coding and theme development, with explicit attention to how researchers’ professional backgrounds and expectations could shape analytical decisions. Field notes capturing contextual observations and initial analytical impressions informed this process by supporting the examination of potential researcher influence on interpretation. These reflexive practices were integrated into consensus discussions on coding and theme refinement. These strategies contributed to analytical credibility by reinforcing that interpretations remained grounded in participants’ accounts. Rather than claiming generalizability, the aim was to provide sufficient contextual transparency for readers to judge the transferability of the findings to other settings.

### Ethical considerations

Ethical approval was obtained from the Research Ethics Committee of Hospital Universitari Arnau de Vilanova (reference number: CEIC-2842) on 22 May 2023. All participants received written information about the study and provided written informed consent prior to participation. Confidentiality and anonymity were ensured throughout the study.

## Results

### Participants

The participants included sixteen women and one man. Participants spent >80% of the working day seated and experienced spinal pain, mainly located in the lumbar and cervical regions. Sociodemographic details are reported more specifically in Table 1.

**Table 1:** Participant characteristics.

Gender	Age	Sedentary time in work (hours)	Type of work	Spinal pain time (in years)	Spinal pain location	Physiotherapy visits
Female	45	7	Administrative and Coordination tasks	> 10	Cervical	No
Female	55	7	Administrative tasks and Public attendance	> 5	Cervical	No
Female	61	7	Administrative tasks	> 20	Dorsal /Cervical	Sporadically
Female	45	7	Administrative and Coordination tasks	> 10	Dorsal Cervical	Sporadically
Female	50	6	Administrative and Commissionaire tasks	15	Cervical	Every 2 weeks
Female	61	6/7	Administrative tasks	> 25	Low back	Sporadically
Female	40	8	Administrative and Coordination tasks	> 5	Low back /Cervical	Every 2 Months
Female	59	7	Administrative tasks	4	Low back /Cervical	Monthly
Female	54	7	Administrative and Commissionaire tasks	> 10	Low back /Cervical	Monthly
Female	61	8	Administrative tasks	20	Low back /Cervical	Sporadically
Female	49	7	Administrative tasks	9	Low back	Sporadically
Female	60	7,3	Administrative tasks and Public attendance	2	Low back	Sporadically
Female	51	7	Administrative tasks	> 10	Cervical	Sporadically
Female	47	7	Administrative and Coordination tasks	> 5	Cervical	No
Female	49	7	Administrative tasks and Public attendance	10	Low back /Cervical	Monthly
Male	47	7	Administrative tasks and Public attendance	15	Low back Cervical	Every 2 Months
Female	56	7	Administrative tasks and Public attendance	> 10	Cervical	No

**Table 2:** Categories and textual quotes for the theme “Work-related spinal pain”.

Categories	Quotes
Pain experiences and beliefs	*"Even if I sit with correct posture, I don’t feel comfortable. I tend to hunch over, and when I stay upright for too long it actually hurts me. Maintaining good posture for even a short time is really hard for me." (P11)* *"I think it’s a constant discomfort, but you get used to it and stop noticing it as much. Still, I would say it’s quite constant." (P14)* *"Well, I think it started as a postural problem, but now, during stressful times, I feel I put pressure on myself, and that’s when the pain gets worse..." (P7)*
Coping pain strategies	*"What’s the solution? Well… nothing really. Just keep working.” (P7)* *"There’s no solution. I just live with the pain… hehehe". (P8)* *"Yeah, when I'm agitated, I do go to a physiotherapist. So I don't end up pushing it too far." (P1)* *"Well, all of that helps, of course, but you can’t always rely on something external, otherwise you’re just giving your power away to it…" (P17)* *"For me, it feels better, not the first two days, but the following days after going… until it builds up again. Maybe… I feel fine for just a week" (P8)*

**Table 3:** Categories and textual quotes for the theme “Implementation of active breaks at work”.

Categories	Quotes
Benefits of exercising in working hours	*"I suppose any kind of movement that relaxes the muscles in the back area. Or sometimes even taking rests, helps, like for the arm, depending on how much the mouse is used, whether extensively or not. What kind precisely? I have no idea." (P4)* *"Afterwards I notice that if I stop and stretch my neck… I feel better." (P2)* *"Yes, well… it has always been said that taking breaks can help you disconnect a bit from what you're doing and relax. And that is… that is good." (P13)*
Difficulties of performing active breaks	*"In my workplace, because we deal with the public, you can’t control when they call you, come through the door, or when the boss calls you to sign something. So others interrupt your break and… you can’t control that.” (P8)* *"Well, I haven’t, but I guess it depends on your boss… they might say no. The effort should start with the supervisors, raising awareness that this benefits the whole institution" (P12)*
Time spent and frequency of performing active breaks	*"Well, yes, to me, 10* min *of disconnection seems maybe a bit too much, but 5* min *every couple of hours while you're working like that. Well, it could be, but I think it's a lot, 5 or 10* min *every hour. I mean, if you do it every two or three hours, that would be enough." (P13)* *"For example, that thing about every hour, that would be, I suppose, what is established, right? It's just that if you are working, when you realise, two hours have passed. And that's not realistic." (P14)* *"Well, it would also be feasible to stop, knowing that you don't have to stop every half an hour, because then the effect would be interrupted. I guess that wouldn't be the expected effect, but to be effective… Everything is manageable, everything is calculable…" (P4)*

**Table 4:** Categories and textual quotes for the theme “Feasibility of a digital intervention in the workplace”.

Categories	Quotes
Media content of the web-based intervention	*"I find the virtual campus convenient… we’re used to using it. You just have to know where to find the information. For work, I think it’s more suitable to use the computer than the phone.” (P1)* *I find a virtual campus more suitable, something on the computer rather than something on the phone." (P1)* *"I believe tutorials are excellent, provided they're concise. We live in a society that thrives on speed, instant gratification, and if the first 10 s don't hook me, I tend to skip. So, they should be intuitive capsules, captivatingly positioned for easy access. Much like FAQs-short, concise, addressing immediate needs, such as 'Does your back hurt here?'… Click." (P7)*
Compliance with the intervention	*"I think what would work best for us as secretaries, in our specific group, is to have a person come in for a set time to explain things. It would force all of us to pay attention without any interruptions from attending to the public during that time. I believe that would work because if I receive an email, I'll look at it, but…" (P15)* *"I think it's more motivating to do it if we all do it at the same time." (P15)* *"I think the method of dissemination the less important thing. I mean, I believe what's most important is creating a plan that's right so that people are motivated and aware, and that's it. I think that's the least of it." (P17)*
Individualization or guidance by a health professional	*"When there are forums, we get lost in the immensity. From experience, just looking at info lists… it has to be very, very directed, person to person..." (P9)* *"Something like a video call or something similar where you can see that person. It doesn't need to be in person, but you should have visual contact." (P10)* *“With a physio it would be ideal. It would be helpful if someone showed us at the beginning which exercises are appropriate for the back… that would be perfect.” (P14)*

### Key themes

Three main themes were identified in the data analysis: Work related spinal pain; Implementation of active breaks in their working routine; and the feasibility of a digital intervention in the workplace.

#### Theme 1: work-related spinal pain (Table 2)

• *Pain experiences and beliefs*

Participants highlighted that maintaining proper posture while working was challenging. Even though they initially aimed for good posture, they tended to relax and adopt less suitable positions over time. Also, not only the correct posture but also too many hours working in the same position aggravates the discomfort.

The workers with spinal pain were used to this discomfort while working; even though the pain was constant, it was not making them stop, being something ordinary in their working days.

Participants discussed how workload and job stress could exacerbate spinal pain. In particular, increased workload on particularly demanding days exacerbates spinal pain symptoms, making discomfort more noticeable throughout the workday.

• Coping pain strategies

Some participants resigned themselves to persistent spinal pain at work, seeing no immediate solutions or remedies considering it a regular part of working while experiencing spinal pain.

Participants shared their experiences managing chronic spinal pain, and visiting a physiotherapist emerged as a prevalent approach. There was a divergence of opinions among participants, with a majority seeking physiotherapy when experiencing pain, with the expectation of pain relief as a normalised practice.

However, a participant expressed that while such interventions help, reliance on external treatments should not overshadow the importance of self-management. Another participant, although visiting a physiotherapist, expressed dissatisfaction as the relief experienced was often short-lived.

#### Theme 2: implementation of active breaks at work (Table 3)

• Benefits of exercising in working hours

When workers were asked about strategies to relieve spinal pain and improve physical well-being; they emphasised that doing any movements, such as mobility and stretching exercises, to prevent stiffness and reduce pain could help reduce discomfort.

Some participants even perform stretches with no prescription just because posture is prolonged and giving movement is good for relieving tension in the spine.

The participants believed that incorporating active breaks into their work routines could yield multiple benefits, extending beyond physical health to encompass mental well-being and productivity. They believed that taking brief breaks allowed them to step away from their primary tasks, engage with different thoughts or activities, and return with a renewed focus.

• Difficulties of performing active breaks

Participants discussed the feasibility of incorporating active breaks in their work routines. While the majority recognised the potential benefits for well-being and job performance, they noted difficulties due to heavy workloads and interruptions. Also, public-facing roles added more complexity.

Another concern raised by participants was the potential for misinterpretation by superiors. They feared that upper management might perceive these breaks as slacking off or unproductive, highlighting proactive efforts to make superiors aware of the potential benefits of active breaks and dispel any misconceptions about their impact on productivity.

• Time spent and frequency of performing active breaks

Participants generally preferred short and frequent active breaks, suggesting the feasibility of taking 3 to 5-minute breaks every two to three hours. They found these brief moments effective for relieving tension and enhancing concentration.

Also, adhering to the recommended practice of taking 10–15 min breaks every hour was unfeasible, as they perceived it as disruptive to their productivity. These extended interruptions were regarded as impractical due to their heavy workload and job responsibilities.

Flexibility was preferred over rigid scheduling. Participants valued the autonomy to decide when and how to take breaks, allowing them to adapt to specific situations and manage their time effectively. This autonomy reduced the sense of obligation and increased acceptance of active breaks.

#### Theme 3: feasibility of a digital exercise intervention in the workplace (Table 4)

• Media content of the web-based intervention

Participants preferred the virtual campus as their primary tool for working because it was easy to use. They recognized the potential of digital tools, particularly the virtual campus, to enhance intervention adoption and success by prioritizing effective communication and accessibility.

Participants preferred short, concise content, similar to the brevity of social media short videos, for effective information delivery, including brief instructional videos with exercises. They emphasized the importance of brevity to maintain engagement. Visual elements like images and infographics were also considered valuable supplements to enhance exercise understanding alongside short videos.

• Compliance with the intervention

Some participants preferred in-person interventions, viewing them as potentially more effective in achieving their desired outcomes than digital alternatives. They believed that the absence of face-to-face interaction could lead to forgetfulness or a lack of willpower, potentially causing them to not adhere to the digital intervention.

Group exercises are viewed positively, fostering community and shared responsibility. These motivate participants' adherence and social interaction while doing exercise at work. Participants consider them effective for establishing healthy routines, particularly for desk-bound individuals seeking benefits for well-being.

Some participants believe that raising awareness about the intervention's importance for their health is the most crucial aspect. They argue that the specific delivery method becomes less important once individuals are genuinely aware of its importance.

• Individualization or guidance by a health professional

Several participants emphasized the importance of receiving individualized guidance within a digital exercise intervention at work. Rather than extensive or generic information, they valued direct and clearly targeted support that could help them feel oriented and accompanied during the intervention. Participants highlighted that overly broad or impersonal digital formats (e.g., forums or large repositories of information) could be overwhelming and reduce engagement.

In this context, guidance from a health professional, including physiotherapists, as a form of support within the app, was perceived as potentially helpful, particularly when delivered through direct and visually mediated contact (e.g., video calls), to enhance trust, clarify doubts, and support adherence. Personalized interaction was considered especially relevant for addressing concerns related to work-related spinal pain and ensuring that the intervention felt meaningful, credible, and applicable to their individual situation.

## Discussion

The primary objective of this study was to explore office workers' perceptions regarding spinal pain at work and their coping strategies. Three themes emerged: work-related spinal pain, implementation of active breaks, and feasibility of a digital intervention. Participants associated pain with posture, ergonomics, and workload, and described short self-managed active breaks as a feasible strategy to reduce discomfort.

Qualitative research has shown that people with chronic spinal pain often develop explanatory models linking their symptoms to work-related demands, posture, workload, and everyday activities.^21^, ^22^, ^23^ Recent qualitative evidence in people with migraine and neck pain also suggests that individuals may attribute neck symptoms to posture and emotional factors and may experience uncertainty regarding appropriate self-management strategies.^24^ In line with this literature, participants in our study described their spinal pain as closely related to their work context and reported having become accustomed to living with pain as something almost inevitable.

Unlike findings from some qualitative studies describing uncertainty about the causes and consequences of low back pain,^21^ participants in our sample demonstrated a clear awareness of the role of their work in the persistence of pain. This more coherent explanatory framework may reflect a less catastrophic interpretation of symptoms, which could facilitate coping and adaptation. Consistent with previous evidence, more negative pain beliefs have been associated with greater pain severity and higher levels of disability in people with low back pain.^25^

The predominance of women in the sample reflects the gender distribution of administrative roles in many workplaces.^26^ Gender-related factors may influence pain perception, coping strategies, and preferences for interventions, which should be considered when designing workplace health programs. Furthermore, epidemiological studies consistently show that women are more likely than men to experience chronic pain, particularly in the cervical and lumbar spine regions. This higher prevalence is linked to biological, psychosocial, and occupational factors, reinforcing the relevance of gender-sensitive approaches in workplace health programs.^27^^,^^28^

Participants tended to rely on passive strategies (physiotherapy or ergonomic adjustments) rather than regular active breaks. This aligns with evidence indicating preference for non-active approaches such as heat, cold or manual therapy over exercise.^22^ These strategies reflect a biomedical focus, while current chronic pain guidelines recommend a biopsychosocial model.^29^ Participants mainly sought help when pain intensified, consistent with Gardner’s review on workplace-related factors.^30^

Within this context, active breaks can be understood as a practical way to operationalize these biopsychosocial recommendations in daily work routines, aligning pain management with the realities of the workplace.

Building on this explanatory framework, participants described mobility and stretching exercises as helpful for relieving tension and maintaining focus during the workday. They viewed these active breaks as alleviating physical discomfort and enhancing concentration, aligning with findings from Waongenngarm’s systematic review^31^ on the benefits of short breaks for physical and mental well-being at work.

However, despite the acknowledged benefits, participants identified substantial impediments to integrating active breaks into their routines. The main challenges were heavy workloads, interruptions, and insufficient managerial support, consistent with barriers described in previous workplace studies.^32^^,^^33^ Interventions should therefore address these organizational constraints and include strategies such as scheduled breaks, suitable spaces, and managerial involvement.

Participants also expressed their preferences regarding active breaks' timing, duration, and frequency. They preferred shorter, more frequent breaks, reflecting the practicality of integrating 3 to 5-minute intervals every two to three hours. This preference aligns with the concept of microbreaks, which have been shown to be effective in reducing musculoskeletal discomfort^34^ and well-being^35^ without substantially disrupting workflow. Moreover, participants were reluctant to perform longer, more disruptive breaks every hour, indicating a perceived trade-off between extended interruptions and productivity. Therefore, the intervention should offer flexibility in break scheduling and duration and tailor them to the participants' needs and preferences.

Digital delivery was perceived as practical for facilitating active breaks, particularly when content was concise and visually accessible, resembling familiar social media formats.^36^ These findings are consistent with previous studies that have reported positive attitudes and intentions of digital interventions for reducing sedentary time.^37^^,^^38^ In this context, individualized guidance may enhance confidence, clarify doubts, and support adherence, aligning with previous research.^39^

### Study limitations

While this qualitative study sheds light on office workers' perceptions regarding spinal pain at work and potential coping strategies, it holds limitations. As a qualitative study conducted within a specific occupational context, the findings offer an in-depth understanding of office workers’ experiences. These results are not intended to be generalized; their potential transferability to other settings should be considered in relation to the contextual features, participant characteristics, and organizational conditions described. Furthermore, the predominance of women in the sample reflects the gender distribution of participants’ roles but may limit the transferability of findings to male workers, as gender-related factors can influence pain perception, coping strategies, and preferences for interventions.

Pain experiences were explored through self-reported accounts, and pain intensity was not systematically quantified, as the qualitative phase did not aim to compare experiences across levels of symptom severity. Variations in perceived pain intensity may have influenced participants’ narratives. In addition, the scope and duration of the interviews were designed to address a focused set of topics within an applied occupational context. This may have limited the exploration of broader or more nuanced aspects of participants’ experiences. Consistent with descriptive qualitative approaches using systematic text condensation, data adequacy was considered in relation to thematic saturation and information power, taking into account the focused study aim, the specificity of the sample, and the consistency of themes across interviews.^15^^,^^18^ Contextual factors within the specific workplace setting could limit the transferability of identified coping strategies and intervention feasibility to other organisational environments.

However, despite these limitations, the study provided valuable insights into the impact of work-related factors on spinal pain, emphasising the potential feasibility of implementing active breaks and digital interventions. These findings suggest that tailored interventions may be needed to address workplace-specific challenges, offering flexibility in break scheduling, and garnering managerial support to ensure successful intervention implementation in diverse work settings.

These findings will inform the subsequent intervention phase of the mixed-methods project, supporting the refinement of digital active breaks and their workplace implementation.

## Conclusion

Office workers acknowledged the perceived benefits of active breaks as coping strategies to alleviate spinal pain and improve focus at work. However, challenges such as heavy workloads, lack of managerial support, and workplace culture hindered their integration. These findings highlight the importance of designing tailored, feasible interventions that align with workers’ expectations, preferences, and daily routines to enhance adherence and effectiveness. The predominance of women in the sample suggests that gender-related factors may be relevant when interpreting these findings in workplace health programs.

Some participants perceived guidance from health professionals, including physiotherapists, as potentially helpful for the development and acceptability of digital active break interventions, which should be interpreted within the context of this qualitative study.

## Declaration of generative AI and AI-assisted technologies in the writing process

During the preparation of this work the principal author used ChatGPT in order to improve language and readability. After using this tool, the authors reviewed and edited the content as needed and take full responsibility for the content of the publication.

## Funding statement

This research did not receive any specific grant from funding agencies in the public, commercial, or not-for-profit sectors.

## Trial and protocol registration

ClinicalTrials.gov Identifier: NCT05571124. Date of registration: 06/10/2022.

## Author contributions

Conceptualisation: Carlos Tersa‑Miralles, Francesc Rubí‑Carnacea, Esther Rubinat Arnaldo. Methodology: Carlos Tersa‑Miralles, Filip Bellon, Maria Masbernat‑Almenara. Investigation: Carlos Tersa‑Miralles, Cristina Bravo, Filip Bellon. Data curation: Carlos Tersa‑Miralles, Cristina Bravo, Maria Masbernat‑Almenara. Writing – original draft: Carlos Tersa‑Miralles Writing – review & editing: Cristina Bravo, Filip Bellon, Maria Masbernat‑Almenara, Francesc Rubí‑Carnacea, Esther Rubinat Arnaldo. Supervision: Francesc Rubí‑Carnacea, Esther Rubinat Arnaldo. Project administration: Carlos Tersa‑Miralles, Francesc Rubí‑Carnacea.

## Patient or public contribution

No patient or public contribution.

## Declaration of competing interest

None.

## Appendix Supplementary materials

Image, application 1

Image, application 2
