# Supplementary material for: Office workers’ perspectives on implementing active breaks supported by a digital intervention to alleviate spinal pain: a qualitative study
Source: Braz J Phys Ther. 2026 Aug 11;30(5):101634. doi: 10.1016/j.bjpt.2026.101634 (PMC13487610; doi:10.1016/j.bjpt.2026.101634)
Supplement: Supplementary file 2 [file mmc2.pdf]

Semi-structured interview script

Interview with administrative staff experiencing back discomfort

Thank you for agreeing to participate in this interview as an administrative staff member at the university. The objective is to understand your opinion on the relationship between spending long hours sitting at work and back care, as well as the implementation of exercises to relieve back pain.

During the interview, we will ask you a series of questions to learn about your opinion and experience regarding back pain at work and possible solutions. We appreciate your collaboration and honesty in your responses, as all the information will be valuable for our study.

To begin, we would like to hear your thoughts on back pain in your work environment...

Back pain:

- What do you think causes your back pain?
- Have you ever experienced back pain in your current job? How would you describe the frequency and intensity of the pain?
- How does back pain affect your work performance? Have you ever had to take time off due to pain?
- Do you think back pain is a common problem among office workers?
- What do you usually do when you have back pain at work? Have you implemented any strategies to prevent or relieve the pain?
- Have you received any treatment for your back pain?

Posture and ergonomics:

- Do you think that sitting in a chair for many hours a day could be related to the back pain you experience?
- Have you noticed any difference in the intensity of your back pain when changing posture at work (for example, standing up or sitting in a different chair)?
- Do you think your workstation's ergonomics are adequate to prevent back pain?

Exercise at work:

Active breaks are short interruptions during the workday to perform exercises or stretches that help reduce muscle tension, fatigue, and stress—common problems associated with sedentary computer-based work.

- Do you think exercising at work could help prevent or reduce back pain?

- Have you ever tried incorporating active breaks into your daily work routine? If so, how much time did you dedicate to them, and how did it go?
- What types of activities do you think would be useful for an active break?
- Do you think it would be easy or difficult for you to incorporate active breaks into your daily work routine?
- Do you think it is possible to do active breaks in short periods, such as 5 to 10 minutes per hour? How often would you like to take them during your workday?
- Do you think the time spent on active breaks could interfere with your workload or productivity during the workday?
- Do you think it would be beneficial to have the support or guidance of a health or wellness professional for performing active breaks?
- What obstacles do you think you might encounter when trying to do active breaks at work, and how could you overcome them?

Use of the Sakai platform:

- Through which medium (YouTube, email, website, etc.) would you prefer to access information and tutorials on active breaks?
- Do you think it would be beneficial to have access to videos or tutorials about active breaks on the Sakai platform, created by a health and exercise professional?
- Do you think implementing active breaks on the Sakai platform would be easy to follow and use?
- What kind of resources or tools would you like to find on the Sakai platform for active breaks?
- Do you think the Sakai platform could help you become more aware of your health at work?
- What obstacles might you encounter in using the Sakai platform for active breaks, and how could you overcome them?
- Would you like to add anything else to the interview?

Guión entrevista semiestructurada

Entrevista a personal administrativo con molestias de espalda

Muchas gracias por aceptar participar en esta entrevista como personal administrativo de la universidad. El objetivo es conocer su opinión sobre la relación entre pasar muchas horas sentado/a en el trabajo y el cuidado de la espalda, así como la implementación de ejercicios para aliviar el dolor de espalda.

Durante la entrevista, le haremos una serie de preguntas para conocer su opinión y experiencia en relación con el dolor de espalda en el trabajo y las posibles soluciones. Le agradecemos de antemano su colaboración y honestidad en sus respuestas, ya que toda información será de utilidad para nuestro estudio.

Para empezar, nos gustaría saber su opinión sobre el dolor de espalda en su entorno laboral...

Dolor de espalda:

- ¿A qué cree que se debe su dolor de espalda?
- ¿Alguna vez ha experimentado dolor de espalda en su trabajo actual? ¿Cómo describiría la frecuencia y la intensidad del dolor?
- ¿Cómo afecta el dolor de espalda su desempeño en el trabajo? ¿Ha tenido que ausentarse del trabajo debido al dolor?
- ¿Cree que el dolor de espalda es un problema común entre los trabajadores de oficina?
- ¿Qué suele hacer cuando tiene dolor de espalda en el trabajo? ¿Ha implementado alguna estrategia para prevenir o aliviar el dolor?
- ¿Ha recibido algún tratamiento por su dolor de espalda?

Postura y ergonomía:

- ¿Cree que estar sentado/a en una silla durante muchas horas al día puede estar relacionado con el dolor de espalda que experimenta?
- ¿Ha notado alguna diferencia en la intensidad del dolor de espalda al cambiar de postura en su trabajo (por ejemplo, al estar de pie o al sentarse en una silla diferente)?
- ¿Cree que la ergonomía de su puesto de trabajo es adecuada para prevenir el dolor de espalda?

Ejercicio en el trabajo:

Teniendo en cuenta que una pausa activa es una práctica que consiste en realizar breves interrupciones durante la jornada laboral para hacer ejercicios o estiramientos

que ayudan a reducir la tensión muscular, la fatiga y el estrés, problemas comunes en trabajos que requieren pasar muchas horas sentados frente al ordenador...

- ¿Cree que hacer ejercicio en el trabajo podría ayudar a prevenir o reducir el dolor de espalda?
- ¿Ha intentado incorporar pausas activas en su rutina diaria de trabajo en el pasado? Si es así, ¿cuánto tiempo ha dedicado a ellas y cómo le ha ido?
- ¿Qué tipo de actividades cree que podrían ser útiles en una pausa activa?
- ¿Cree que sería fácil o difícil para usted incorporar pausas activas en su rutina diaria de trabajo?
- ¿Cree que es posible realizar pausas activas en períodos cortos de tiempo, como de 5 a 10 minutos cada hora? ¿Con qué frecuencia le gustaría hacerlas durante su jornada laboral?
- ¿Cree que el tiempo dedicado a las pausas activas podría interferir en su carga de trabajo o en su productividad durante la jornada laboral?
- ¿Cree que sería beneficioso contar con la ayuda o la orientación de un profesional de la salud o del bienestar para realizar pausas activas?
- ¿Qué obstáculos cree que podría encontrar para hacer pausas activas en el trabajo y cómo podría superarlos?

Uso de la plataforma Sakai:

- ¿A través de qué medio (YouTube, correo electrónico, página web, etc.) le gustaría tener acceso a la información y a los tutoriales para realizar las pausas activas?
- ¿Cree que sería beneficioso para usted tener acceso a vídeos o tutoriales sobre pausas activas en la plataforma Sakai realizados por un profesional de la salud y el ejercicio?
- ¿Cree que la implementación de pausas activas en la plataforma Sakai sería fácil de seguir y de utilizar?
- ¿Qué tipo de recursos o herramientas le gustaría encontrar en la plataforma Sakai para realizar pausas activas?
- ¿Cree que la plataforma Sakai podría ayudarle a ser más consciente de su salud en el trabajo?
- ¿Qué obstáculos podría encontrar para utilizar la plataforma Sakai para realizar pausas activas y cómo podría superarlos?
- ¿Le gustaría añadir algo más a la entrevista?
